# Correction to “Prevalence, Recovery and Phenotype of Dysphagia in Patients With Ischaemic Cerebellar Stroke”

**DOI:** 10.1111/ene.70806

**Published:** 2026-11-10

J.H. Schaefer , F. Luft , A. Seiler , et al., “Prevalence, Recovery and Phenotype of Dysphagia in Patients With Ischaemic Cerebellar Stroke,” European Journal of Neurology 31, no. 7 (2024): e16303, 10.1111/ene.16303.PMC1123585638634169

In the published version, Reference 11 was inadvertently omitted in two sentences in the Discussion section and in one sentence in the Conclusion section. The citation should be added immediately after the following sentences:

“In addition, patients presenting with persisting dysphagia after isolated cerebellar stroke should be carefully evaluated for additional involvement of the brainstem, as isolated cerebellar damage might not be sufficient to cause severe and persisting dysphagia.”

“One plausible reason as to why damage to the cerebellum alone may not result in overt dysphagia is owed to the representations of the swallowing muscles occurring in multiple locations over the cerebellum.”

“In contrast to lesions in the cerebral cortex, or the brainstem, damage to a localized part of the cerebellum appears to be less deleterious to the swallowing network.”

We apologize for this error.
